# COVID-19 Vaccine Hesitancy and its Reasons in Addis Ababa, Ethiopia: A Cross-Sectional Study

**DOI:** 10.4314/ejhs.v32i6.2

**Published:** 2022-11

**Authors:** Tamrat Assefa Tadesse, Ashenafi Antheneh, Ashenafi Teklu, Asres Teshome, Bemnet Alemayehu, Alemu Belayneh, Dessale Abate, Alfoalem Araba Abiye

**Affiliations:** 1 School of Pharmacy, College of Health Sciences, Addis Ababa University, Addis Ababa, Ethiopia; 2 Department of Pharmacy, College of Health Science, Debre Berhan University, Debre Berhan, Ethiopia

**Keywords:** Coronavirus, COVID-19 vaccine, vaccine hesitancy

## Abstract

**Background:**

Coronavirus disease (COVID-19) vaccine hesitancy becomes the major bottleneck to the global healthcare system in minimizing the spread of the virus. This study aimed at assessing COVID-19 vaccine hesitancy and its reasons among residents of Addis Ababa, Ethiopia.

**Methods:**

A community-based cross-sectional survey was conducted between May 16 to 29, 2021 in purposively selected four districts of Addis Ababa, Ethiopia. A structured questionnaire was developed and then designed on Google Forms platforms to collect data from study participants after obtaining a verbal consent form. A total of 422 study participants were included in the survey. Data were entered into Microsoft Excel and then exported to the Statistical Package for the Social Sciences (SPSS) version 25 for analysis.

**Results:**

Face masks and alcohol hand rub/ sanitizer are used by 50. 7 and 24.9% of respondents when required. COVID-19 was thought to have been generated by humans by a substantial percentage of study participants (38.2%). About half (50.7%) and 24.9% of respondents use face masks and alcohol hand rub/sanitizer always when it is required, respectively. A large number of study participants (38.2%) believed that origin of COVID-19 is man-made. Overall, 242 (57.4%) of study participants reported COVID-19 vaccine hesitancy. Fear of vaccine side effects (49.6%) was the most common reason for hesitancy. Doubt about its effectiveness (33.9%), not having enough information about the COVID-19 vaccine, preferring another way of protection, and unreliable of the vaccine (due to its short development period) were also the most frequently mentioned reasons for not receiving the COVID-19 vaccine.

**Conclusions:**

COVID-19 vaccine hesitancy rate was high in Addis Ababa, Ethiopia during the study period. Fear of side effects, doubts about its effectiveness, and not having enough information about the COVID-19 vaccine were major reasons for hesitancy. Continuous awareness creation to the community on the importance of vaccination is warranted by health professionals and healthcare cadres.

## Introduction

Coronavirus disease 2019 (COVID-19) is a global pandemic caused by a virus called severe acute respiratory syndrome coronavirus 2 (1). The disease continued to be a major threat to the public causing morbidity and mortality all over the world. According to the World Health Organization (WHO) report as of March 03. In 2022, there were 442,423,496 confirmed cases of COVID-19 with 6,001, 938 deaths throughout the world (2).

COVID-19 preventive measures include using a facemask, proper handwashing, maintaining physical distance, and vaccination, which helps to minimize pandemic transmission (3). Vaccines have long been a reliable method of disease prevention, but their hesitancy concern continues to be an obstacle to widespread vaccination of highly contagious diseases like COVID-19. It is necessary to achieve high COVID-19 vaccination acceptance rates to achieve a large coverage of population immunity but, vaccine hesitancy poses a serious challenge in this quest (4). According to some studies conducted in the United States, Europe, and China, vaccine safety is a global concern (5). Vaccine preventive action is essential in protecting the overall public health of the population (6). Higher levels of human immunity limit the ability of viruses to spread pandemics, and it would be achieved by the wide use of an effective vaccine (7).

Vaccine hesitancy refers to a delay in acceptance, or refusal of vaccines despite the availability of vaccine services. The term encompasses outright refusal to vaccinate, delaying vaccines, accepting vaccines but remaining uncertain about their use, or using certain vaccines but not others. Vaccine hesitancy is complex and context-specific, varying across time, place, and vaccines. It is influenced by factors such as complacency, convenience, and confidence. It has been linked to religious values, personal beliefs, and safety issues based on widespread misconceptions, such as the connection between vaccines and autism, brain injury, and other disorders, according to various reports (8).

According to Machingaidze and Wiysonge, “Vaccine hesitancy is pervasive, misinformed, contagious, and is not limited to COVID-19 vaccination” (5). Evidence showed that there is a conspiracy belief within the public that “COVID-19 vaccines are intended to inject microchips into recipients and that the vaccines are related to infertility”. Those individuals having lower health literacy, employees, females, non-whites, religious beliefs, psychiatric conditions, lower incomes, and education levels are more likely to believe in conspiracy theories and the percentage of the supporter ranges from 23–56% (9–12). According to a survey conducted in Jordan and Kuwait, more than 25% of the respondents had a conspiracy belief that COVID-19 vaccines are intended to inject microchips into recipients and could cause infertility (9).

Experts recommend that 60% of the population vaccination is mandatory to ban the transmission of this global pandemic. But this amount of vaccination may be difficult because of vaccine hesitancy in society. This data shows that it needs extra effort to get the vaccine and to reduce vaccine hesitancy to have better COVID-19 vaccine coverage in the nation (9). Among many factors which contributed to decreased vaccination coverage against COVID-19, hesitancy toward vaccination may take the lead in low-income countries (4). However, little is known about its magnitude in the capital city of Ethiopia, Addis Ababa. Therefore, this study was designed to assess the COVID-19 vaccine hesitancy rate, reasons for hesitancy, and its determinants among residents in Addis Ababa, Ethiopia during the early initiation of the COVID-19 vaccine.

## Methods

**Study setting**: This study was conducted in Addis Ababa, the capital city of Ethiopia. Addis Ababa has a population of 4,794,000, 11 sub-cities, 116 districts, and an area of 527 km^2^ making the population density estimated to be near 5,165 individuals per square kilometer available.

**Study design and period**: A community-based cross-sectional survey was conducted in Addis Ababa, Ethiopia between May 16 to 29, 2021 among adult individuals who live in four districts of four sub-cities in Addis Ababa, Ethiopia.

**Inclusion and exclusion criteria**: Individuals who were 18 years old and above, who live within the selected four districts (1 district from each sub-city), and volunteers were included in the study. Exclusion criteria were being healthcare professionals and those who already received the COVID-19 vaccine during the data collection period.

**Sample size determination and sampling techniques**: A sample size (n = 422) was determined using a single population proportion formula, by taking a 95% confidence interval, 5% margin of error, 50% proportion of vaccine hesitancy, and adding up a 10% non-response rate. After selecting four districts purposively, the online survey link was shared with data collectors with the purpose of the study and consent form. To prevent information saturation, only one family member who fulfilled the inclusion criteria was selected by the lottery method if there was more than one member during data collection.

**Data collection tool and procedures**: Data were collected using a structured questionnaire developed from a comprehensive literature review (13–15), and then designed on Google Forms platforms. The questionnaire has a total of 32 items which were grouped into 4 sections: socio-demographic, general questions on COVID-19 and personal health, COVID-19 vaccine-related questions; and attitude and perception towards COVID-19 and its vaccine. Conspiracy beliefs about COVID-19's origin, willingness to take the COVID-19 vaccine when available, belief that COVID-19 vaccines are a biological weapon, it could cause infertility, the vaccine intends to make money and the vaccine itself could cause COVID-19 were assessed by using vaccine conspiracy belief Likert scale(15–20). The online questionnaire was used to interview residents with help from the assigned data collectors.

**Data quality assurance**: The survey questionnaire was developed initially in the English language and translated to Amharic and then back to English to maintain its consistency. The Amharic version was used for data collection. A pre-test was conducted on 10% of the sample size. After the pre-test, necessary amendments were made before the commencement of data collection. A half-day training was given to data collectors on how to handle Google Forms platform questionnaires, understandability of questions, and how to communicate with study participants. Moreover, regular supervision, spot-checking, and reviewing of the completed questionnaire were carried out daily to maintain data quality.

**Data analysis**: The response obtained from Google Forms was downloaded as a Microsoft Excel file and then all variables were filtered and replaced by equivalent English translation. Data coding, cleaning, and verification were also performed before computing analysis using statistical package for social sciences (SPSS) version 25 software. Descriptive statistics including frequency, percentage, mean (standard deviation) and median (interquartile range) were used to summarize sociodemographic and other descriptive data.

**Ethical consideration**: The study was conducted according to the guidelines of the Declaration of Helsinki and approved by the School of Pharmacy Associate Dean's Office of Addis Ababa University (Ref No: AD/039/20/13/21). All participants provided verbal informed consent before participating in the study. The study participants were informed that the information they provided was handled with strict confidentiality.

## Results

**Socio-demographic characteristics**: Out of 422 study participants, nearly half (49.3%) were females and twelve of them were pregnant. The mean age was 28.93 ± 11.13 with a range of 18 to 72 years. Most of the study participants (70.4%) were in the age group of 18 to 29 years old. In this study, 71.1% and 69.9% were single and Orthodox by religion, respectively. About a quarter of respondents reported that there was at least one healthcare professional living with them. The mean family size was 5.1 ± 2.*28* (Table 1).

**Table 1:** Sociodemographic profile of study participants, Addis Ababa, Ethiopia

	Variables	N	%
Sex	Male	214	50.7
	Female	208	49.3
Age in years	18–29	297	70.4
	30–49	87	20.6
	50–72	38	9
Marital status	Single	300	71.1
	Married	104	24.6
	Others a	18	4.3
Religion	Orthodox	296	70.3
	Protestant	73	17.3
	Muslim	47	11.2
	Others b	5	1.2
Educational status	Degree and above	241	57.1
	Diploma	81	19.2
	Secondary school (grade 9 -12)	66	15.6
	Primary school (grade 1 -8)	16	3.8
	Able to read and write	13	3.1
	Unable to read and write	5	1.2
Employment status	Student	150	35.5
	Government employee	140	33.2
	Self-employed	94	22.3
	Others c	38	9.1
Monthly income (ETB)	No income	82	19.4
	< 1000	70	16.6
	1001 -4999	123	29.2
	> 5000	147	34.8
With whom do you live?	Family	344	81.5
	Alone	64	15.2
	University/camp	14	3.3
Family size in number (N = 337)	2 -5	214	60.5
	> 6	123	36.4
Is there a health professional living	Yes	108	26.1
with you? (N = 414)	No	306	73.1

a widowed, divorced, separated

b catholic, Buddhism, atheists

c non-governmental organization, not working

**General health and COVID-19-related information**: Most of the study participants (89.6%) reported as they do not have any known chronic disease. Personal and/or family history of COVID-19 infection was reported by 27.5% of the study population. Television/radio and social media were the main sources of information about COVID-19 and its vaccine as indicated by 63.2% and 50.7% of study participants, respectively (Table 2).

**Table 2:** General health and COVID-19 related information of study participants.

Variables		N	%
Do you have any known chronic diseases?	Yes	44	10.4
	No	378	89.6
How do you perceive your health status?	Very good	237	56.2
	Good	148	35.1
	Average	32	7.6
	Bad	4	0.9
	Very bad	1	0.1
Have you or any of your family members had COVID-19?	Yes	116	27.5
	No	306	72.5
How often do you use a face mask when required?	Always	214	50.7
	Often/usually	149	35.3
	Occasionally	54	12.8
	Never	5	1.2
How often do you use an alcohol hand rub/sanitizer when it is required?	Always	105	24.9
	Often/usually	157	37.2
	Occasionally	141	33.4
	Never	16	3.8
How often do you maintain your physical distance when required?	Always	51	12.1
	Often/usually	131	31.0
	Occasionally	182	43.1
	Never	58	13.7
What is your belief about the origin of the COVID-19 virus?	Natural sources	113	26.8
	Man-made virus	161	38.2
	I do not know	148	35.1
What is your main source of information about COVID- and its vaccine?	TV/Radio	266	63.0
	Social media	214	50.7
	Healthcare professionals	95	22.5
	Newspaper	33	7.8
	Others*	33	7.8

* Includes responses like I do not have any source of information, random individuals, colleagues/friends, scientific journals, or personal search from the websites

**Figure 1:**
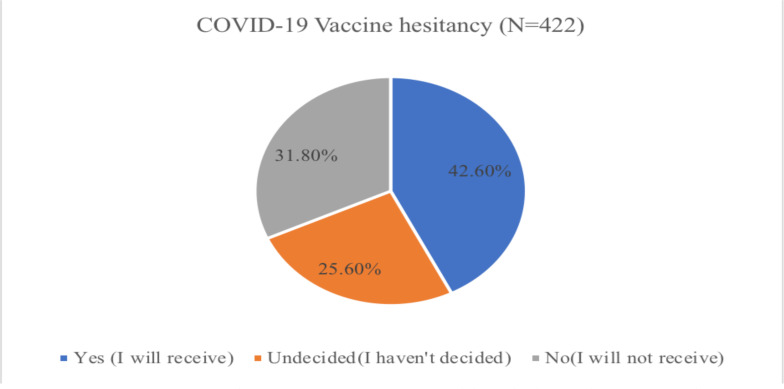
COVID-19 vaccine hesitancy rate among Addis Ababa, Ethiopia residents.

COVID-19 vaccine hesitancy: Out of the total, 242 (57.4%) of study participants hesitated (will not receive (No) or were undecided to receive) the COVID-19 vaccine when it is available to them (Figure 1).

**Figure 2:**
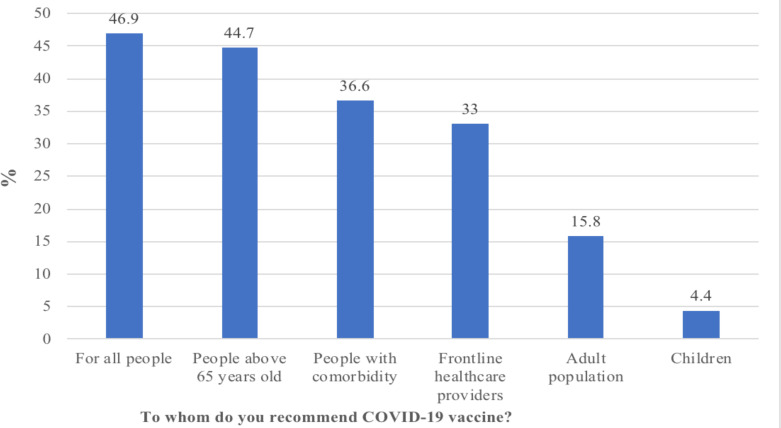
Study participants' COVID-19 vaccine recommendation to other individuals

In this study, 63% of participants responded that they recommend the COVID-19 vaccine to others. The most mentioned recommendation was for all people (46.9%) and people above 65 years old (44.7%) and the least recommendation was for children (4.4%) (Figure 2).

**Figure 3:**
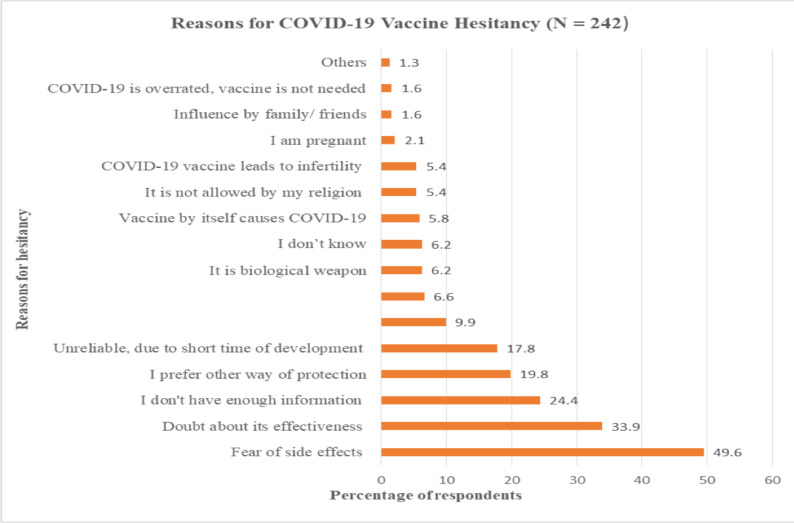
Reasons for COVID-19 vaccine hesitancy of study participants, Addis Ababa, Ethiopia.

**Reasons for COVID-19 vaccine hesitancy**: Out of 242 respondents who hesitated to receive the COVID-19 vaccine when it is available, nearly half of them (49.6%) reported fear of vaccine side effects as a reason for not taking and 33.9% doubted its effectiveness. Not having enough information about the COVID-19 vaccine, preferring another way of protection, and unreliable of the vaccine (due to short time development) were also the most frequently mentioned reasons for not receiving COVID -19 vaccine (Figure 3).

Attitudes and Beliefs about COVID-19 and its Vaccine: Overall, 192 (45.5%) strongly agree that COVID-19 is a real disease. Only 13% strongly agree that they will feel safe after vaccination. Ninety-one (21.6%) replied that they strongly worry about the serious unknown effects of the COVID-19 vaccine (Table 3).

**Table 3:** Attitude/Belief toward COVID-19 and its vaccine

Attitude/Belief description	Strongly agree N (%)	Agree N (%)	Neutral N (%)	Disagree N (%)	Strongly disagree N (%)
COVID-19 is a real disease	192 (45.5)	127 (30.1)	36 (8.5)	33 (7.8)	34 (8.1)
COVID-19 is a new disease, vaccine have not been fully tested	79 (18.7)	144 (34.1)	126 (29.9)	61(14.5)	12 (2.8)
I feel safe after being vaccinated	55 (13.0)	162 (38.4)	135 (32.0)	52 (12.3)	18 (4.3)
Although most vaccines are safe, there may be problems with COVID-19 vaccines	97 (23.0)	205 (48.6)	76 (18.0)	37 (8.8)	7 (1.7)
I worry about the series unknown effects of COVID-19 vaccine in the future	91 (21.6)	165 (39.1)	116 (27.5)	43 (10.2)	7 (1.7)
Authorities promote vaccines for financial gain not for people	29 (6.9)	43 (10.2)	179 (42.4)	146 (34.6)	25 (5.9)
A vaccination program is beneficial	127 (30.1)	180 (42.7)	83 (19.7)	25 (5.9)	7 (1.7)
Being exposed to disease naturally is safer for the immune system	81(19.2)	148 (35.1)	103(24.4)	74 (17.5)	16 (3.8)

## Discussion

COVID-19 pandemic became a major challenge since its declaration as a global pandemic and different preventive mechanisms were tried to halt this pandemic. Vaccination was one of the best preventive measures to prevent transmission of the COVID-19 pandemic. Recent evidence showed that to stop the spread of the COVID-19 pandemic and to develop herd immunity, 60–70% of society should be vaccinated (13). Therefore, the highest acceptance of the COVID-19 vaccine has a greater role to control the worldwide COVID-19 pandemic. But the main challenge is the presence of vaccine hesitancy.

COVID-19 vaccine hesitancy had been recognized as a global challenge by WHO especially in low and middle-income countries (LMICs) (14). Most of the current study participants were students in the age range of 18–29 years. Individuals within this age group can easily access information through various social media outlets and are highly vulnerable to falsified and biased information(23–25)

In this study,57.4% (will not receive and undecided to receive) of the study participants were hesitant to receive the COVID-19 vaccine. The result is higher than the hesitancy rate reported in many studies conducted in different parts of Ethiopia (17,20,26–29) and online survey in Sub-Saharan countries(30). However, a higher COVID-19 vaccine hesitancy rate (60.3%) was documented among healthcare workers in Addis Ababa, Ethiopia (31) in patients with chronic disease visiting health facility in Northeastern Ethiopia and African Americans(34%). Furthermore, the hesitancy rate in this study is higher than the result This is an alarming figure when compared to the international vaccine hesitancy rate and it is challenging to achieve the required herd immunity (60–70%) to stop the spread of the COVID-19 pandemic with this considerable hesitancy rate (7).

This low acceptance of vaccination may be due to the belief that the COVID-19 virus is human-made. This conspiracy belief has infiltrated the attitude of people about the COVID-19 pandemic around the globe. People have misconceptions about the origin and prospective vaccines' safety and efficacy (21,32,33). We must combat disinformation by aggressively disseminating factual information about the realities of COVID-19 and the risks and benefits of immunization to promote vaccine adoption. Furthermore, rather than dismissing people's worries regarding the COVID-19 vaccine, we must acknowledge them. Furthermore, we must moderate public expectations and inform the public that participants in the trials leading to emergency use permission ranged in age, racial and ethnic backgrounds, and comorbidities. Healthcare professionals must anticipate, validate, and be ready to respond to people's questions and concerns, and they must show their confidence that the benefits of vaccination outweigh the dangers by being vaccinated as soon as the opportunity arises (25,34).

In this study, the main reason for COVID-19 hesitancy was the fear of side effects (49.6%) followed by skepticism about the effectiveness of the COVID-19 vaccines. Concerns about vaccine safety and efficacy have been frequently highlighted in other studies. (35–37). This highest fear of safety and efficacy could be due to the negative attitude of society towards the vaccine which could be related to the conspiracy beliefs about the origination of the virus and the intention of the vaccine (9). On the other hand, vaccine development takes years of preclinical and clinical trials, but the COVID-19 vaccine was developed quickly, which could be one of the causes for concerns regarding the vaccine's immediate and long-term safety and efficacy.. (38).

In the current study, nearly one-fourth of the hesitant study population claimed that they didn't have enough information on COVID-19 and its vaccine. This indicates that misunderstandings about the disease's origins and vaccination intentions may have a strong impact on COVID-19 vaccine acceptance. Conspiracy theories may be falsely believed as a result of misinformation from various social media outlets, religious beliefs, or peer advice (14,23,24). Hence, a structured and well-prepared factual communication by the government and responsible public media is vital to preclude society from misleading conspiracy theories and anti-vaccine information which contributes to the burden of morbidity and mortality from confirmed COVID-19 cases.

There are some limitations to this study. First, the data was gathered from only four districts in Addis Ababa and did not represent the city's entire population's vaccine hesitancy rate and its reasons. Another limitation is that participants' hesitancy to vaccines may change over time as new vaccine shipments arrive and more people are vaccinated, which may affect their initial hesitancy toward vaccination.

Finally, we summarize that the COVID-19 vaccine hesitancy rate was higher. Many reasons were identified for hesitating to receive the COVID-19 vaccine when it is available for study participants. Continuous awareness creation a health professional is needed regarding the safety, efficacy, and unclear conspiracy belief of society. The media and other stakeholders should work together to build public trust by disseminating accurate and consistent vaccine information. A nationwide study is recommended to address the scope of COVID-19 hesitancy and to implement a solution at the Ministry of Health level to attain the target population herd immunity.
